# Influence of the Dermis Thickness on the Results of the Skin Treatment with Monopolar and Bipolar Radiofrequency Currents

**DOI:** 10.1155/2016/1953203

**Published:** 2016-07-14

**Authors:** Ilja L. Kruglikov

**Affiliations:** Scientific Department, Wellcomet GmbH, 76229 Karlsruhe, Germany

## Abstract

Electrically layered tissue structure significantly modifies distribution of radiofrequency (RF) current in the dermis and in the subcutaneous adipose tissue comparing to that in a homogeneous medium. On the basis of the simple model of RF current distribution in a two-layer skin containing dermis and subcutis, we assess the influence of the dermal thickness on the current density in different skin layers. Under other equal conditions, current density in the dermis is higher for the skin having thinner dermis. This contradicts the main paradigm of the RF theory stating that treatment results are mainly dependent on the maximal temperature reached in a target tissue, since the best short- and long-term clinical results of RF application to the skin were reported in the areas having thicker dermis. To resolve this contradiction, it is proposed that the long-term effect of RF can be realized through a structural modification of the subcutaneous fat depot adjacent to the treated skin area. Stimulation of these cells located near the interface dermis/subcutis will demand the concentration of applied RF energy in this area and will require the optimal arrangement of RF electrodes on the skin surface.

## 1. Introduction

Application of radiofrequency (RF) current to the skin was supposed to be able to modulate its mechanical properties and consequently to improve the skin laxity. Theoretically, this effect was connected with a Joule heating of the dermis leading to conformational changes in its collagen network (so-called “collagen shrinkage” effect) as well as with stimulation of collagen production* de novo* induced by elevated temperature [1].

Improvement of the facial skin structure after application of RF current was demonstrated in different clinical studies [1–4], although the observed results varied in different facial areas. For example, the main outcome reported in [2] after application of monopolar RF current was the improvement in moderate cheek laxity as well as in nasolabial and mesolabial folds; at the same time, the submandibular and the upper neck skin laxity demonstrated significantly worse improvement after the same treatment. Thus, different facial subareas in the same subject demonstrate various improvements after the same RF treatment.

Peculiarities of the RF current distribution in the skin are determined by different external parameters, among them the type and the spatial arrangement of RF electrodes, as well as by some internal characteristics of the target area and by electrical characteristics of the pathways from the electrodes to the target. Whereas the external parameters can generally be reliably controlled, the internal characteristics can demonstrate strong intersubject and interareal variations. One important internal property of the skin significantly influencing the RF current distribution is its layered structure with distinct interfaces between the media having different electrical characteristics [5].

From electrical point of view, most important interfaces are the stratum corneum/viable skin and the dermis/subcutaneous white adipose tissue (sWAT). The next interface sWAT/muscle is normally far away from the interface dermis/sWAT and its influence on the current distribution in the dermis can be neglected in the first approximation. Such skin structure should cause the concentration of the current in the tissue having the highest electrical conductivity (dermis) and reduce its penetration into the sWAT which is much more electrically resistive [5]. This effect can even dominate over the peculiarities of the current distribution produced by different spatial arrangement of the electrodes on the skin surface, thus effectively diminishing the influence of external parameters on the treatment outcome.

Supposing that the local temperature elevation is the main reason for observed mechanical skin modulation after RF current application and taking into account that this elevation is quadratic dependent on the local current density [6], the impact of RF current on the skin should be improved for the configuration of RF electrodes providing the optimal current densities in a predefined target structure. Such optimal arrangement of RF electrodes taking account of the layered skin structure can substantially deviate from the corresponding configuration of the same electrodes placed on the surface of a homogeneous medium and it is very different from configurations usually used in clinical applications [5]. Nonoptimal configuration of RF electrodes can reduce the current density in the target area several times, thus significantly decreasing the desired heating effect.

Dermis thickness (DT) is the universal scale parameter of electrically layered skin [5]. All other spatial characteristics of the system, for example, distance between the electrodes, can be measured in the units of DT. It can be assumed that variations of DT can strongly influence the current distribution and are mainly responsible for the observed interarea and intersubject variations of clinical results. The main purpose of this paper was to analyze how the DT variations can influence the current distribution in the skin and to compare these theoretical results with outcomes of RF current applications to the body areas having different DT values.

## 2. Variations of the Facial Dermis Thickness

Information about regional, sexual, and age-dependent variations of the DT is contradictive. It is known that the absolute values of DT are dependent on the measurement procedure being different* in vitro* and* in vivo*, whereas the DT values determined* in vitro* were claimed to be bigger than the corresponding values measured* in vivo* [7]. It was also shown that DT in the same body area can significantly vary with age and degree of photodamage [8–10].

Facial skin thickness in adult cadavers demonstrates strong spatial variations being in average bigger in the cheek and chin areas and smaller in the neck [11]. For example, in [11], the DT in the neck varied in the range (0.25 mm, 0.80 mm), while it varied in the range (0.57 mm, 1.62 mm) in malar eminence and (1.04 mm, 1.20 mm) in the cheek area. Much more minutiae measurements in 45 cadavers (27 male and 18 female) provided the thicknesses of 0.95 ± 0.28 mm for the nonwrinkled areas of the skin, whereas the whole range of measured values was (0.35 mm, 1.65 mm) [12]. The values of DT in the wrinkle locations in the same subjects were 0.81 ± 0.31 mm with the range of (0.12 mm, 1.74 mm). Contrarily to these results, the measurements of the skin thickness with 20 MHz ultrasound* in vivo* provided the average DT values of about 1.6 mm in the cheek area and more than 2.5 mm in the chin [9, 10].

The intersubject variations for the same facial subareas are also high. According to [11], the individual DT values in the area of malar eminence measured in three cadavers were 0.97 ± 0.07 mm, 1.62 ± 0.05 mm, and 0.57 ± 0.04 mm, correspondingly. The average DT for all three subjects was 1.05 ± 0.45 mm, which demonstrates that the high coefficient of variation obtained in this study was mainly caused by strong intrasubject variations. At the same time, coefficient of variation for the DT profile in the same facial subarea was sufficiently lower, being for single subjects about 3–7%. Thus, in a first approximation, the variations of the skin thickness profile inside of the same facial subarea can be neglected, but these variations should be taken into account if the different subareas in one subject or if different subjects are considered. If such variations of DT can significantly influence the current distribution and the corresponding temperature elevation in the skin, the absolute DT values in a given facial area should correlate with observed effectiveness of RF treatment.

## 3. Effect of DT on RF Current Distribution in the Skin

To determine how the current distribution in a layered skin depends on DT, we will first consider the monopolar current electrode placed on the skin surface. Since the* stratum corneum* layer of the skin is very thin, its influence on the current distribution in the dermis will be neglected. Skin is a lossy dielectric [6], and thus the electric potential $\varphi \left(\vec{r},t\right)$ produced in an electrically layered structure can be found as a solution of the Poisson equation taking into account the dielectric properties as well as the polarization of the interfaces between the layers. To solve this problem analytically, an approximation in which this polarization is neglected will be considered; this approximation takes into consideration radiofrequencies for which the skin is predominantly electrically conductive. Hence, the conductive currents in single skin layers must be much larger than the corresponding displacement currents, that is, *σ*(*f*) ≫ 2*πfε*_0_ *ε*(*f*), where *f* is the current frequency, *σ*(*f*) is the electrical conductivity at frequency *f*, *ε*_0_ is the free space permittivity, and *ε*(*f*) is the relative permittivity of the tissue at frequency *f*. This reduces the Poisson equation to the Laplace equation [5]:

(1) $$\begin{array}{c} \nabla \left(\;\sigma \left(\;\vec{r},f\;\right)\nabla \varphi \left(\;\vec{r},t\;\right)\;\right)=0, \end{array}$$

where $\vec{r}$ is the radius-vector and $\sigma \left(\vec{r},f\right)$ is the local electrical conductivity of the target tissue which depends on the current frequency, *f*. Further we will consider the skin as a two-layer structure with a plane, isotropic, homogeneous boundary dermis/subcutis located parallel to the skin surface. Although such model is a simplification, it gives possibility to analyze the influence of DT on the current distribution in the skin.

The local current density, $\vec{J}\left(\vec{r}\right)$, can be found from the following equation:

(2) $$\begin{array}{c} \vec{J}\left(\;\vec{r}\;\right)=-\sigma \left(\;\vec{r}\;\right)\vec{\nabla }\varphi =\sigma \left(\;\vec{r}\;\right)\vec{E}\left(\;\vec{r}\;\right). \end{array}$$

Equation (1) can be easily solved for the point electrode placed on the top of a layered medium in an integral form written in cylindrical coordinates [13]. In this geometry, every point in the skin can be described by the set of three parameters {*r*, *z*, *ψ*}, where *r* is the radial distance from RF electrode, *z* is the depth into the skin, and *ψ* is the azimuth. If the point monopolar current source is placed on the polar axis (*r* = 0) and the adjacent medium can be considered as isotropic, the distribution of potentials will be independent of *ψ*. Electric potentials *φ*_*dm*_ in the dermis and *φ*_*sm*_ in the sWAT produced by a monopolar point RF electrode placed on the skin surface and delivering the total current *I*_0_ into the skin can be presented in cylindrical coordinates in integral forms [5]:

(3) φdmr,z=I02πσd1z2+r2+k∫0∞e−2αd1−ke−2αdeαz+e−αzJ0αrdα,

(4) φsmr,z=I02πσd1z2+r2+k∫0∞1−e−2αd1−ke−2αde−αzJ0αrdα,

where *d* is the thickness of the dermis; *k* is the reflection coefficient of the current at the interface dermis/sWAT, *k* = (*σ*_*d*_ − *σ*_*s*_)/(*σ*_*d*_ + *σ*_*s*_); *σ*_*d*_ and *σ*_*s*_ are the electrical conductivities of dermis and sWAT, respectively; and *J*_0_ is the Bessel function of order zero. In (3) and (4), index *d* refers to dermis, *s* to subcutis, and *m* to monopolar current, respectively.

From (3) and (4), distribution of electric potential in the skin is dependent on the reflection coefficient *k*, which vary with morphological structure and physiological state of the dermis and sWAT and which is a dispersive parameter. For example, for RF current of *f* = 1 MHz, the electrical conductivity of the viable skin is about *σ*_*d*_≅0.4 S/m [14], whereas the average electrical conductivity of sWAT is about *σ*_*s*_≅0.02 S/m [15]. Thus the “physiological” value of *k* for this interface is about 0.905 [5]. Increase of *σ*_*s*_ two times by the same value of *σ*_*d*_ will reduce *k* to approximately 0.800; reduction of *σ*_*s*_ two times will increase *k* up to 0.950.

From (2) and (4), the radial, *J*_*smr*_^*lay*^, and vertical, *J*_*smz*_^*lay*^, components of the current density in sWAT can be presented as

(5) Jsmrlayr,z−σs∂φsm∂r=I0r2π1−k∑n=0∞knr2+2nd+z23/2,

(6) Jsmzlayr,z−σs∂φsm∂z=I02π1−k∑n=0∞kn2nd+zr2+2nd+z23/2.

In *J*_*smr*_^*lay*^ and *J*_*smr*_^*lay*^, indexes *r* and *z* refer to the radial and vertical components of the current density and index *lay* refers to the layered skin structure. Corresponding components of the current densities in a homogeneous medium are

(7) Jsmrhomr,z=I02πrr2+z23/2,Jsmzhomr,z=I02πzr2+z23/2.

To compare the vertical components of the monopolar RF current at the same depth *z* under the electrode (*r* = 0) in sWAT in the layered and homogeneous skins, we will consider the following ratio:

(8) $$\begin{array}{c} \alpha_{sm}\left(\;0,z\;\right)=\frac{J_{smz}^{lay}\left(0,z\right)}{J_{smz}^{hom}\left(0,z\right)}=\left(\;1-k\;\right)\overset{\infty }{\underset{n=0}{\sum }}k^{n}\frac{z^{2}}{{\left(2nd+z\right)}^{2}}. \end{array}$$

At *k* = 0.905, the ratio of current densities in the layered and homogeneous skin at *z* = *d* (corresponding to the location of the interface dermis/sWAT) is *α*_*sm*_ = 0.112. Since *J*_*smz*_^*lay*^(0, *d*) describes the RF current crossing the interface dermis/sWAT and entering subcutis at *r* = 0, it can be concluded that, under “physiological” conditions (*k* = 0.905), the current distribution near the interface dermis/sWAT is so modified that approximately 9 times less RF current will enter sWAT under the monopolar RF electrode in a layered skin than in a homogeneous medium. At *k* = 0.95,0.8,0.6, this ratio will be *α*_*sm*_ = 0.060,0.227,0.435, respectively. It is seen that deviation of the current distribution in the layered skin from its distribution in a homogeneous medium rapidly increases with *k*.

## 4. Effect of DT on RF Current Density at the Interface Dermis/sWAT

To assess the influence of DT on the RF current density at the interface dermis/sWAT, let us consider the bipolar configuration of RF electrodes on the skin. Electric potential produced by bipolar electrodes is the sum of potentials from two monopolar electrodes, taking into account that the potentials produced by single electrodes in a bipolar configuration have the opposite signs.

Radial component of the bipolar current density in the dermis at the depth *z* under the skin for DT = *d* can be found from (5):

(9) Jdbrlay=Jdbrhom1+L2+4z23/2·∑n=1∞kn1L2+42ndi+z23/2+1L2+42ndi−z23/2,

where *J*_*sbr*_^*lay*^ and *J*_*dbr*_^*hom*^ are the current densities in the layered and homogenous skin, respectively, and index *b* refers to a bipolar current. From (9) it can be easily seen that, for the fixed distance between the electrodes *L* and at the fixed depth *z* under the skin, the local current density in the layered skin quickly reduces with *d*. Thus, the thinner dermis should demonstrate the higher concentration of RF current comparing to the thicker one.

Let us now compare the radial components of RF current densities at the interface dermis/sWAT for the dermis of a single (DT = *d*) and double (DT = 2*d*) thickness. From (9), the ratio of the current density at this interface in the skin having the thickness *d* to corresponding current density in the skin having the thickness 2*d* is 0.158,0.546,0.763 for *L* = *d*, 10*d*, 100*d*, respectively. Thus, with increased distance between the RF electrodes, the influence of DT on the current distribution near the interface dermis/sWAT will be reduced. However, for the small distance *L*, corresponding to the optimal electrodes' configuration providing the highest possible current density [5], the influence of DT is very strong.

Next we will consider the fraction of the RF current which transverses the interface dermis/dWAT. As it was shown in [5], for monopolar RF electrode, in electrically homogeneous medium (*k* = 0), 50% of the RF current flows into the sWAT through the circle of the radius $r_{0}^{hom}=\sqrt{3}d$. In a layered tissue with reflection coefficient of *k* = 0.905 this radius should be approximately *r*_0_^*lay*^ = 23.5*d*. That means that the entrance of 50% of RF current into the sWAT in the layered skin will be distributed over the surface which is approximately 184 times bigger than the corresponding surface in a homogeneous medium. In other words, RF current in a layered medium is strongly redistributed and enters sWAT not directly under the RF electrode, but far away from it. Importantly, the characteristic radius of the surface collecting RF current is proportional to DT. Doubling of DT value will increase the surface collecting the same amount of RF current four times, thus significantly reducing the heating effect near the interface. Additionally, the characteristic radius of surface collecting RF current is strongly dependent on the reflection coefficient and on the RF configuration (monopolar or bipolar).

## 5. Discussion

Distribution of RF current in the skin is dependent on its electric layered structure and can significantly deviate from the corresponding distribution in a homogeneous medium. Two internal physical parameters of the skin which can strongly influence this distribution are (1) dermis thickness and (2) current reflection coefficient at the interface dermis/sWAT, which describes the difference in electrical properties of two adjacent media.

Variations of DT can significantly modulate the current distribution in the dermis as well as its penetration into the sWAT. For example, in the case of the skin having the thicknesses of 1 mm and 2 mm and for the same distance between the RF electrodes of* L* = 10 mm, the ratio of the current densities in thicker/thinner skin at the interface dermis/sWAT in our model will be approximately 0.546. Since the local temperature elevation is proportional to the square of the current density, the induced temperature at this point in the thicker skin will be only 29.8% of its value in the thinner one. This clearly demonstrates that in electrically layered skin RF current is significantly more concentrated in the thinner dermis than in the thicker one and must consequently produce stronger heating in a thinner skin. This result seems to be paradoxical, since it does not confirm the positive correlation between the effectiveness of RF applications in different facial areas and their DT values observed in clinical studies. Indeed, cheek area having bigger DT was reported to demonstrate better reaction to the same amount of RF current than the neck or forehead areas having the thinner dermis [2].

One possibility to resolve this confrontation would be to suppose that not the local current densities (and thus not the local temperatures), but rather the total heated volume (whereas with a lower average temperature in the case of the thicker dermis) is primarily responsible for the clinical results observed short-term after RF applications. Supposing that the clinically observed effect of RF current on the skin is connected with a volume modulation of the dermis, we can speculate about the biophysical mechanism which could be primarily involved in this process. Generally, two components of the skin can be responsible for its quick volume modulation, since only they occupy significant portions of this tissue. The first component is the dermal collagen, which by high temperatures can change its volume through denaturation (shrinkage) or through increase of its amount (collagen production* de novo*). This mechanism was criticized in [16]. The second component of the skin which can quickly react to RF current is the water whose content is strongly dependent on the local concentration of glycosaminoglycans and especially on hyaluronan (HA). It is known that already the mild hyperthermia of about 42°C can significantly increase the production of HA in a target tissue [17]. Such endogenous production of HA will lead to a local water accumulation in the dermis. Indeed, it was shown that the porcine reticular dermis reacts to the application of RF current with a short-term edema building [18]. This effect will be manifested in increase of the skin turgor, which can explain the improvement in the skin texture immediately after RF treatments. Such modification of the skin structure should be observed by significantly lower temperatures than those which are needed for collagen shrinkage.

Whereas quick HA accumulation can explain the short-term results observed after RF treatments, this effect cannot be responsible for any long-term clinical results which were also claimed. Potential target which can be involved in the long-term improvement of mechanical parameters of the skin is the sWAT, especially its superficial layer. This special fat depot contains the adipocytes which have the ability to quickly change their number and volumes [19] and thus can sufficiently influence the skin appearance. Adipocytes from this layer can quickly react to the application of different physical factors [20–22].

Reaction of sWAT to RF currents should be generally connected with a modification of the extracellular matrix in sWAT containing different collagen structures. Electrical conductivities of collagens are much higher than the electrical conductivity of triglycerides filling the adipocytes and occupying the main volume of sWAT. This difference in electrical conductivities will lead to a concentration of RF current in relatively thin collagen networks located around (pericellular fibrosis) or between (intercellular fibrosis) single adipocytes [23]. Such concentration of RF current will provide sufficiently high current densities in collagen structures of sWAT even in the case where the main part of RF current will be reflected and only a small part of it will cross the boundary dermis/subcutis, as it was described in the model above.

Very recently it was shown that the anatomical structures of the adipose tissue in different facial fat compartments can vary significantly [24]. For example, the labial fat compartment characterized by a “fibrous” type of sWAT contains the small groups of mature adipocytes embedded in a dense collagen matrix; the malar compartment, having a “structural” type of sWAT, contains the lobules of mature adipocytes homogeneously covered by thin collagen fibres. Although this question was not investigated systematically, there are some indications that the local dermis thickness correlates with the structure of the adjacent sWAT. So a thicker dermis in the labial area correlates with a “fibrous” type of WAT in the adjacent sWAT compartment. On the other hand, a thinner dermis in the malar area correlates with a “structural” type of the adjacent sWAT depot. Since sWAT of the “fibrous” type contains significantly more fibrotic structures than the “structural” sWAT, the labial area should demonstrate less heating in the dermis; however a stronger heating in adjacent sWAT should lead to the reinforcement of fibrotic structures in this tissue and thus to the change of the mechanical properties and appearance of adjacent skin. This mechanism can resolve the contradictions between the biophysical effects of RF currents and the observed clinical results; however, it clearly shifts the targets for RF currents in aesthetic applications from the dermis to the extracellular matrix in sWAT.

Recently, it was shown in a contralateral design that a pretreatment with RF current improves the efficiency and longevity of HA-based fillers by midface rejuvenation [25]. This correlates with the theory proposed in [26], which explained the long-term effects of the soft tissue fillers by stimulation of proliferation and differentiation of adipose-derived stem cells as well as by local modification of the adipose tissue structure.

Thus it can be assumed that a long-term skin improvement observed after RF application to the skin is connected with a local structural modification of sWAT induced by RF current. This effect should be strongly dependent on the RF current density near the interface dermis/sWAT, which will define the part of RF current penetrating the sWAT. At the same time, the threshold temperatures over 60°C which were supposed to be sufficient for the long-term clinical results after RF applications based on the theory of collagen shrinkage are not needed for the structural modification of the sWAT structure. This is indirectly supported by clinical observations that the treatment of the same facial area with low RF energy applied in multiple passes can provide even better results than application of high RF energy in a single pass [27].

## 6. Conclusion

Electrically layered tissue structure significantly modifies the current distribution in the dermis and sWAT by both monopolar and bipolar application of RF current. Since the dermis thickness significantly varies in different facial areas, this effect must lead to strongly inhomogeneous spatial distribution of the current density. Such current inhomogeneity will lead to even more significant inhomogeneity in induced temperature field. This effect contradicts the main paradigm of the RF theory according to which the treatment results are mainly dependent on the maximal temperature in a target tissue, since the best short- and long-term clinical results of RF application were reported in the areas with the thickest dermis. To resolve this contradiction, we propose that the main short-term effect of RF application is connected with accumulation of hyaluronan and water in the dermis, which must make the effect of RF current on the skin much less temperature dependent as it was assumed before. It is further supposed that the long-term effect of RF is realized through structural modification of the subcutaneous fat depot adjacent to the treated skin area.

Variations of DT can significantly influence the current distribution and thus the temperature profiles in the dermis and sWAT. To provide the structural modification of adjacent sWAT depot, the RF energy should be optimally concentrated at the interface dermis/subcutis. Such optimization is mainly dependent on the configuration of RF electrodes. Taking into account that DT can vary 4–8 times between different facial areas, it is very doubtful that the optimal full-face RF treatment can be provided with a single fixed configuration of RF electrodes.
